# Myositis Ossificans Traumatica of Bilateral Sternocleidomastoid Muscles After Chiropractor Adjustment: A Case Report

**DOI:** 10.7759/cureus.56931

**Published:** 2024-03-26

**Authors:** Martin Felix, Ryan Denis, Charles Chen, Ana Picaza, Damian Casadesus

**Affiliations:** 1 Internal Medicine, St. George’s University School of Medicine, St. George, GRD; 2 Internal Medicine, Jackson Memorial Hospital, Miami, USA

**Keywords:** ectopic ossification, soft tissue masses, myositis ossificans traumatica, heterotopic bone, myositis ossification

## Abstract

A woman in her 20s with a past medical history of surgical debulking of a right neck mass presented to the hospital for persistent and worsening right shoulder pain. The shoulder pain was associated with trismus and back and neck pain. A CT scan of the neck with contrast revealed post-surgical changes with increased heterotopic ossification throughout the surgical site extending to the supraclavicular soft tissues and the left sternocleidomastoid muscle, suggesting muscle ossification. A biopsy was performed, and the patient was diagnosed with myositis ossificans (MO). Initial treatment began with the administration of steroids and analgesics. She was scheduled for a follow-up with orthopedics, rheumatology, and genetics, but she was lost for follow-up. MO is a very rare medical condition usually associated with trauma, and in our patient, the symptoms started after a chiropractic adjustment.

## Introduction

Myositis ossificans (MO), or osteogenic fibrous dysplasia, is a benign medical condition characterized by the abnormal formation of heterotopic bone within skeletal muscles. It is a rare phenomenon, and its etiology involves a mixture of genetic, environmental, and cellular factors. It is divided into MO progressive and MO traumatica. MO progressive is an autosomal dominant disease with bone formation in systemic muscle, fascia, and tendons within a family [1].

In MO traumatica, ectopic ossification typically arises in response to trauma or injury to the affected muscle tissue, triggering an inflammatory cascade that leads to the formation of bone in the muscular compartments [1]. Clinically, individuals with MO traumatica often present with symptoms such as pain, swelling, and restricted joint movement, making accurate diagnosis crucial for appropriate management [1-3]. We report a case of MO arising at the neck of a young female after presenting to the ED with an expanding neck mass. We believe a contributing factor may have been a cervical neck adjustment by a chiropractor.

## Case presentation

A woman in her 20s with no significant past medical history presented to the hospital with worsening shoulder pain and a rapidly enlarging right neck mass. Four months prior, the patient noted a right neck mass that started a few days after a cervical adjustment at the chiropractor. In the emergency room, the vital signs were within normal limits. On the physical examination, cardiopulmonary and abdominal examinations were normal. The patient had a large, hard, palpable mass in the bilateral sternocleidomastoid muscle with no palpable lymphadenopathy, thyromegaly, or trachea midline deviation. Oropharyngeal cavity examination was limited due to severe trismus and limited mouth opening. The patient was admitted to the intermediate care unit for airway monitoring and started on dexamethasone 8 mg IV every 8 hours.

**Figure 1:**
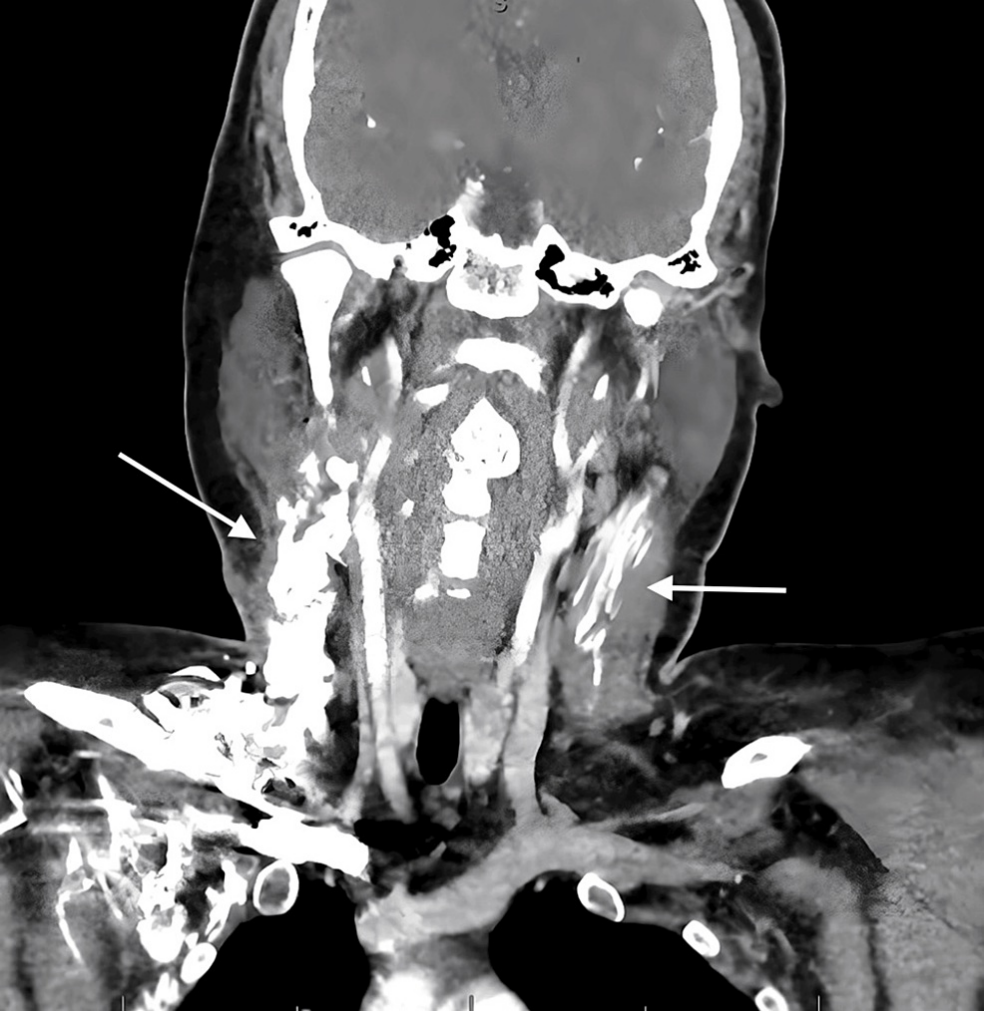
CT scan of the neck with heterotropic bone formation in bilateral sternocleidomastoid muscle

**Figure 2:**
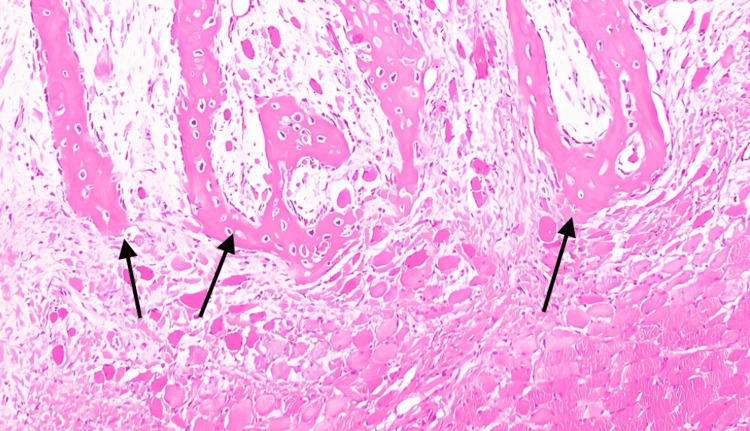
Heterotropic bone formation in the muscle biopsy

The initial CT scan of the neck revealed a possible hematoma and muscle calcifications. The patient underwent a right neck exploration, and the biopsy revealed nodular fasciitis. Despite the continuation of treatment and the reduction of dexamethasone to a daily dosage for a week, the patient’s neck mass remained and was accompanied by worsening trismus (mainly impacting her ability to consume a liquid diet), along with back and neck discomfort. As a result, a right neck dissection was performed 15 days later. A computed tomography (CT) scan of the neck with contrast revealed post-surgical changes with increased heterotopic ossification throughout the surgical site extending to the supraclavicular soft tissues (Figure 1). A large soft tissue density lesion centered in the left sternocleidomastoid muscle suggested muscle ossification. The patient underwent a biopsy of the right neck mass. The biopsy revealed that more than 95% of the lesion was composed of reactive woven bone rimmed by osteoblast, and the residual spindle cell component was largely replaced by heterotrophic bone, suggesting the diagnosis of MO (Figure 2). The patient was discharged home with follow-up with rheumatology, genetics, and orthopedics, but she was lost to follow-up. The patient was followed up in the outpatient clinic for six months and then was lost for follow-up in our hospital.

## Discussion

MO is generally thought of as an ossifying soft-tissue mass. Pathophysiology is not completely understood, but it is likely the result of the inappropriate differentiation of cells in response to an inflammatory environment. It is a self-limiting condition that follows a traumatic injury in 60-80% of cases and occurs in the skeletal muscle of the extremities in about 80% of those cases. Horseback riders and football players are among some of the more commonly affected, with flexor surfaces of the arms and extensors of the legs being the most affected [4]. Our patient has no family or previous personal history of MO progressiva. We suspect the development of MO in our patient can be traumatica. We believe that there may be a correlation between the symptoms experienced after the procedure and chiropractic adjustment. However, we are unable to establish a direct link between the intensity of this trauma and the occurrence of this rare medical condition.

After the initial chiropractor appointment, the patient started noticing pain, stiffness, and a growing mass lasting longer than expected from a simple muscular injury. Although literature varies slightly, MO is reported to progress through three stages: early, intermediate, and mature. The early phase occurs 1-4 weeks after injury and is characterized by an inflammatory state and release of cytokines that act on skeletal muscle, leading to cell differentiation. The intermediate phase, weeks 4-8, presents the secretion of osteoid matrix. During this phase, calcification may begin showing on imaging [3,5,6]. The mature/late phase presents mature bone formation for months until the bone consolidates. In time, bone regression often occurs. During the second admission, our patient presented in the late phase of her condition.

MO can mimic other soft tissue diseases, making diagnosing difficult in the early phases. Our patient was previously diagnosed with nodular fasciitis, probably because the spindle cell component in MO is morphologically identical to nodular fasciitis. Laboratory and image studies have an important role in the diagnosis [7]. Imaging and pathology should be carefully observed to diagnose MO clearly while ruling out other soft tissue conditions such as sarcoma.

There are few reported cases of MO traumatica in the cervical region. Baysal et al. [8] and Lee and coworkers [9] described a patient with traumatic MO of the paraspinal muscle after a blunt trauma with a hard object to the side of the neck and after acupuncture, respectively. Another reported case described the occurrence of MO traumatica of the levator scapula muscle due to neck strain while performing construction work [10]. Read-Jones and coworkers described a patient like ours with traumatic MO in the anterior side of the neck. Their patient developed MO in the belly of the omohyoid muscle after a trauma [10]. To our knowledge, our patient is the first reported case that had both sternocleidomastoid muscles affected, who unfortunately was lost to follow-up while in the process of completing treatment and further clinical workup.

## Conclusions

MO is a condition resulting in bone-producing lesions that are typically self-limiting and post-traumatic. It is usually associated with trauma, and in our patients, the symptoms started after the chiropractic adjustment. Our patient underwent a previous debridement of the mass and was diagnosed with nodular fasciitis. It is probably related to the identical morphology of the spindle cells in both medical conditions. The similitude of the MO and the nodular fasciitis regarding the differentiation of the spindle cells. Physicians should consider MO in the differential diagnosis of a mass formation after trauma.
